# Invasive Cutaneous Mucormycosis in a Patient With Chronic Lymphocytic Leukemia on Obinutuzumab, Idelalisib, and Bruton Tyrosine Kinase Degrader: A Case Report

**DOI:** 10.7759/cureus.92967

**Published:** 2025-09-22

**Authors:** Chelsea D Morinishi, Claire E Brown, Ramee Younes

**Affiliations:** 1 Department of Medicine, Division of Infectious Diseases, David Geffen School of Medicine, University of California, Los Angeles, Los Angeles, USA

**Keywords:** biologics, bruton tyrosine kinase degrader, cutaneous mucorales, idelalisib, immunocompromised, invasive fungal infection, obinutuzumab

## Abstract

Invasive fungal infections (IFIs) due to *Mucorales* are a significant infectious complication in patients with hematologic malignancy, contributing to high mortality rates. The emergence of new targeted biologic therapies has introduced additional effects on the innate and humoral immune system, which produce variable risk for IFIs. Timely diagnosis and treatment of invasive mucormycosis can be challenging. We present a case of invasive cutaneous mucormycosis in an elderly man with chronic lymphocytic leukemia (CLL), who was receiving a Bruton tyrosine kinase (BTK)-targeted protein degrader trial drug (BGB-16673), obinutuzumab, a newer anti-CD20 monoclonal therapy, and idelalisib, a phosphoinositide 3-kinase inhibitor. While molecular testing and tissue culture were negative, histopathology was essential to early diagnosis. Clinical cure was achieved with limb amputation, given the extent of disease. This case underscores the necessity of a low index of suspicion for mucormycosis on presentation, critical appraisal of the patient’s risk factors, and a multimodal approach to diagnosis.

## Introduction

Though rare, invasive mucormycosis is a fungal infection with high mortality, with a pooled estimate of 57% based on a recent meta-analysis on pulmonary diseases [1]. This represents an improvement in mortality rates compared with studies published before 2000, which estimated a pooled mortality rate of 72.1% (p = 0.00001) [1]. Mucorales cause pulmonary, rhino-orbital-cerebral, cutaneous, and disseminated disease. Those with disseminated disease are at higher risk for mortality than those with isolated disease [2]. Immunocompromised patients, such as those with hematologic malignancies, are at the highest risk for disseminated and pulmonary mucormycosis due to several factors, including prolonged neutropenia and corticosteroid use. Conversely, rhino-orbital-cerebral disease is more common in patients with poorly controlled diabetes mellitus. An accurate estimate of the incidence of infection remains elusive, largely due to limitations in the yield of diagnostic methods. Such limitations may contribute to a delay in diagnosis and further contribute to morbidity and mortality. The diagnostic yield of traditional culture methods is known to be low at approximately 50% [3]. Polymerase chain reaction (PCR) based methods on tissue and bronchoalveolar lavage (BAL) specimens have improved pooled sensitivity and specificity (86.4% and 90.6% vs. 97.5% and 95.8%), but may be limited by sample collection technique or tissue preservation [4]. Further, new targeted biologic therapies have expanded treatment options for hematologic malignancies, but little is known about the infectious risks of trial drugs such as Bruton tyrosine kinase (BTK)-targeted protein degraders. We present a case of invasive cutaneous mucormycosis in a patient with chronic lymphocytic leukemia (CLL) on several targeted biologic therapies.

## Case presentation

A 74-year-old male, a retired real-estate agent with a history of CLL, left lower extremity leukemia cutis, and hypothyroidism, presented with two weeks of worsening right ankle swelling and ulceration. With no preceding trauma, a pruritic vesicle had appeared on the ankle, which progressively enlarged. To ease his discomfort, he used a needle to puncture the vesicle, noting purulent drainage. Over two weeks, he developed worsening swelling and ulceration of the wound, and the affected area darkened to a purple hue. He received multiple short courses of antimicrobials, including trimethoprim-sulfamethoxazole, with no improvement. He had no fever, chills, shortness of breath, abdominal discomfort, or other lesions. His active treatment regimen for CLL included obinutuzumab, idelalisib, and a BTK degrader trial drug (BGB-16673). He regularly tended to his plants while wearing open-toed shoes. There was no significant travel history preceding his presentation.

**Figure 1:**
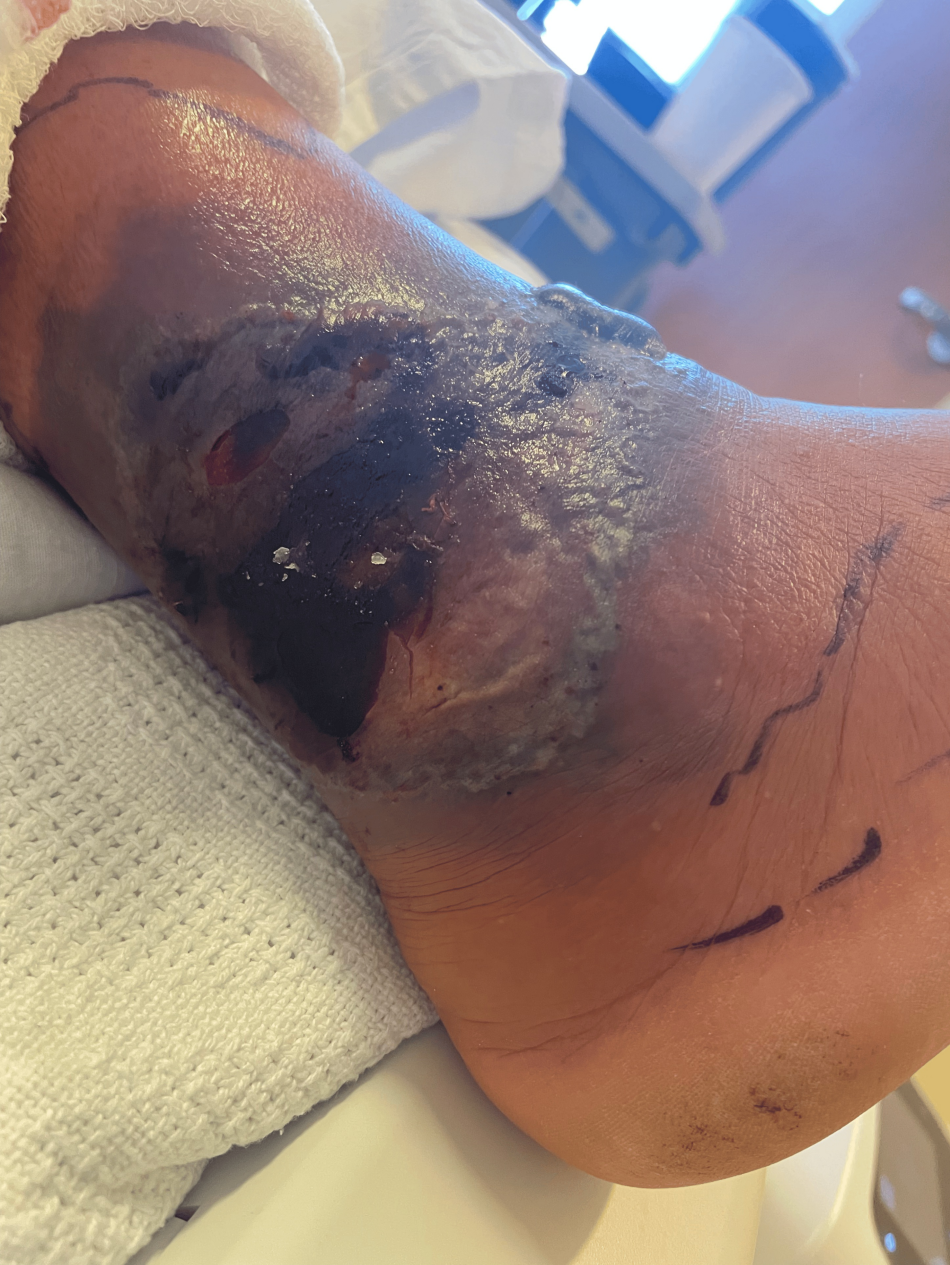
Right ankle ulcer with necrosis and swelling.

**Figure 2:**
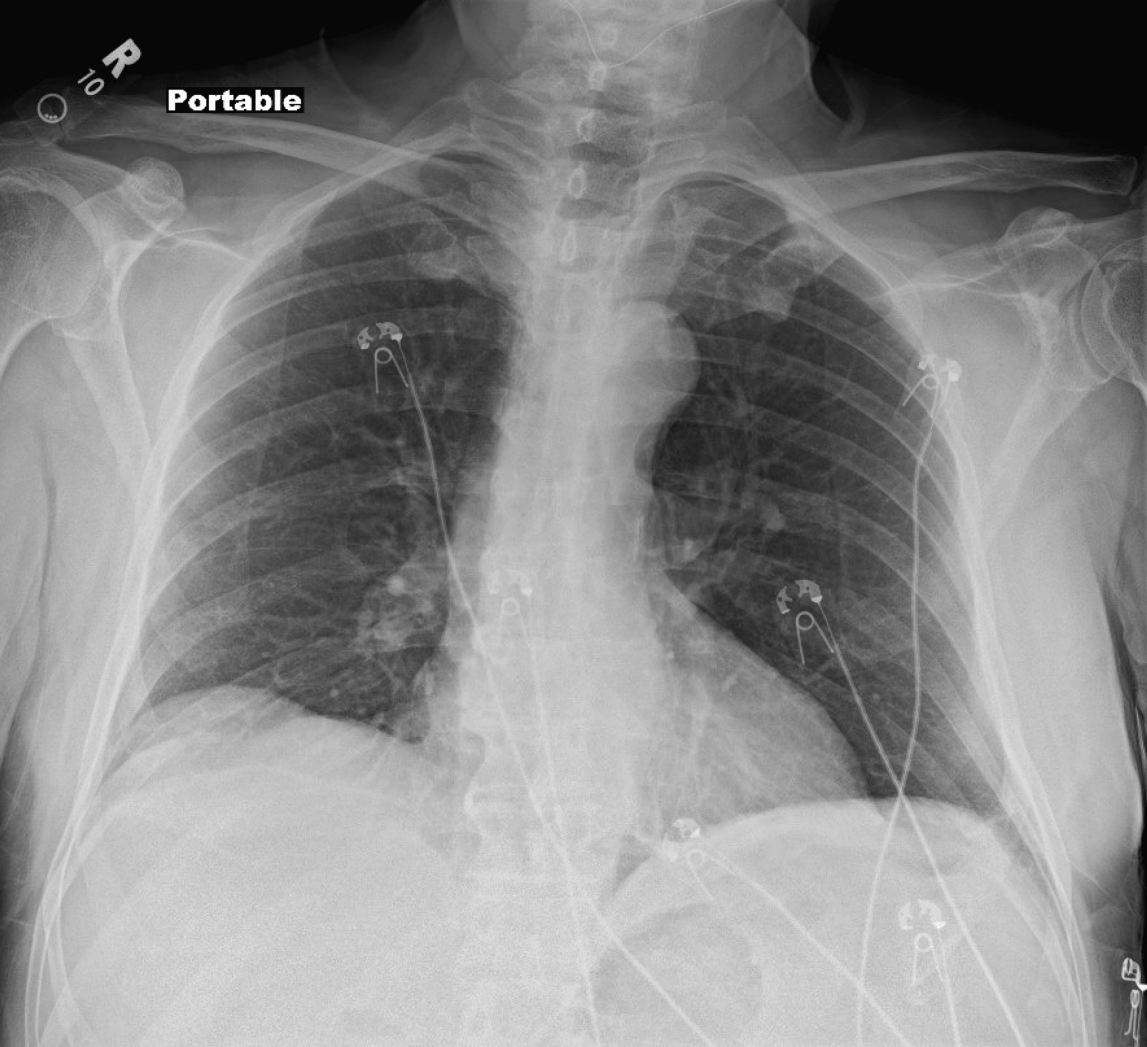
Chest radiograph showing mild right lower lobe scarring without focal consolidations.

**Figure 3:**
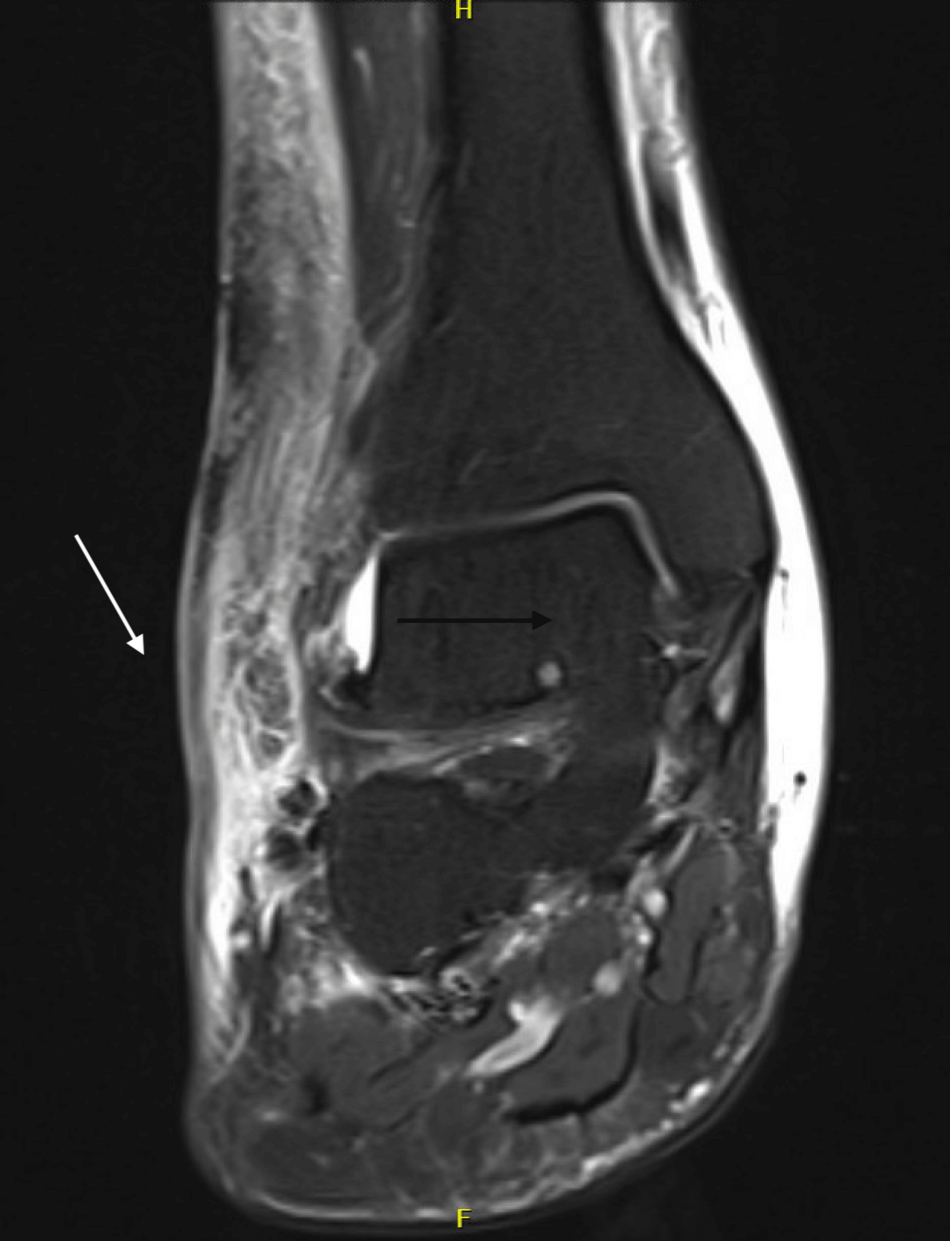
Coronal view of right ankle MRI without contrast showing superficial soft tissue edema and ulceration along the lateral ankle without osteomyelitis.

On examination, he was afebrile with stable vital signs. Skin examination revealed a violaceous plaque with central eschar on the right lateral ankle and tense bullae on the right anterior ankle (Figure 1). No distal lymphadenopathy was appreciated. Laboratory evaluations (Table 1) showed an elevated white blood cell count with lymphocyte predominance, consistent with underlying CLL, and elevated C-reactive protein. Chest radiograph showed mild right lower lobe scarring without focal consolidations (Figure 2). An MRI of the right lower extremity without contrast demonstrated superficial soft tissue edema and ulceration along the lateral ankle without osteomyelitis (Figure 3). He was started empirically on piperacillin-tazobactam and vancomycin.

**Table 1:** Laboratory evaluations.

Parameter	Value	Reference range
White blood cell count	187.29 x 10^3^/uL	4.6 – 9.95 x 10^3^/uL
Absolute neutrophil count (ANC), manual	15.0 x 10^3^/uL	1.8 – 6.9 x 10^3^/uL
Absolute lymphocyte count (ALC), manual	170.4 x 10^3^/uL	1.3 – 3.4 x 10^3^/uL
Hemoglobin	8.7 g/dL	13.7 – 17.5 g/dL
Platelet	278 x 10^3^/uL	163 – 369 x 10^3^/uL
C-reactive protein (CRP)	4.3 mg/dL	<0.8 mg/dL
Erythrocyte sedimentation rate	11 mm/hr	<12 mm/hr
Creatinine	1.11 mg/dL	0.60 – 1.30 mg/dL
Alanine transaminase (ALT)	17 u/L	7 – 52 u/L
Aspartate transaminase (AST)	9 u/L	13 – 39 u/L
Total bilirubin	0.34 mg/dL	0.1 – 1.00 mg/dL
Hemoglobin A1c	5.5%	<5.7%
HIV 4^th^ generation	Negative	Negative

**Figure 4:**
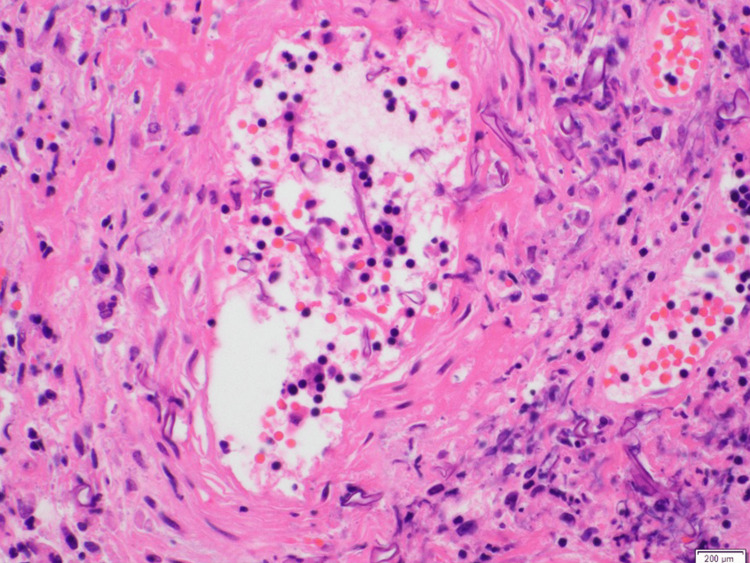
Right ankle tissue with hematoxylin and eosin (H&E) stain at 40x magnification demonstrating fungal hyphae with angioinvasion.

**Figure 5:**
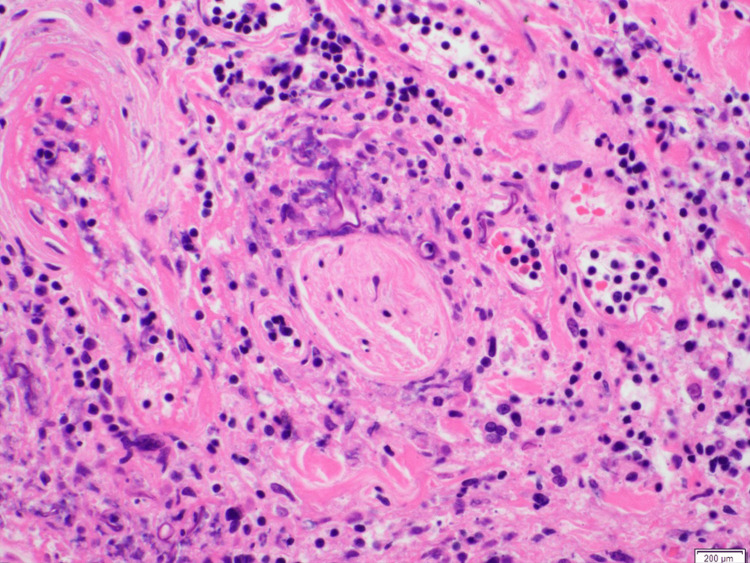
Hematoxylin and eosin (H&E) stain at 40x magnification demonstrating fungal hyphae with perineural invasion.

**Figure 6:**
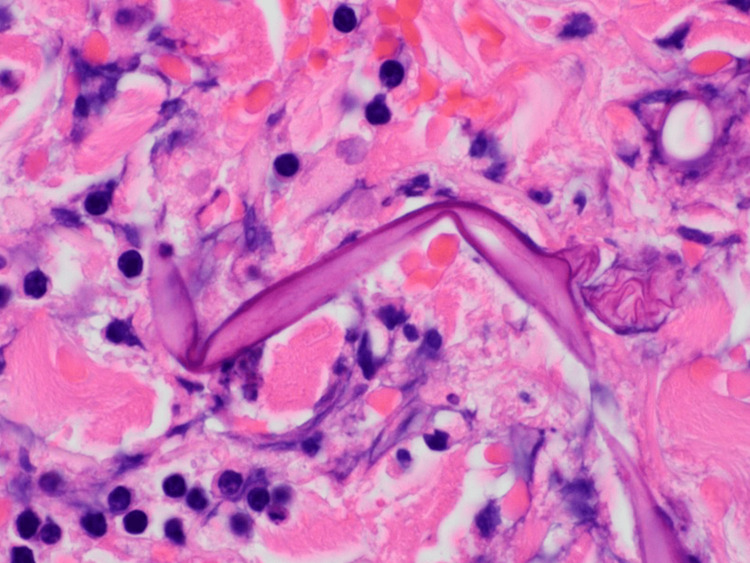
Hematoxylin and eosin (H&E) stain at 100x magnification demonstrating broad, aseptate hyphae.

A punch biopsy of the right lateral ankle ulcer was performed on day two of his hospitalization. The following day, the ulcer appeared to have enlarged. Preliminary pathology findings revealed epidermal and dermal necrosis with scattered, broad aseptate fungal hyphae and angioinvasion (Figures 4-6), pathognomonic for cutaneous mucormycosis. Liposomal amphotericin B (L-AmB) 5 mg/kg daily and micafungin 100 mg daily were initiated. While awaiting fungal, acid-fast bacilli, and bacterial tissue cultures, tissue was sent to the University of Washington for fungal next-generation sequencing (NGS). Prompt surgical debridement revealed extensive necrosis of the underlying fascia, muscle, and tendons, necessitating limb amputation below the knee for definitive source control. L-AmB and micafungin were discontinued after three days, and the patient was transitioned to isavuconazole (ISAV) after surgery. Clean margins were confirmed on histopathology. Concordant with the operative tissue cultures, fungal NGS showed negative results after two weeks. Given no evidence of disseminated infection and clinical stability on outpatient follow-up, ISAV was discontinued six weeks after surgery. Timely diagnosis with histopathology was essential to ensuring clinical cure, as fungal cultures and molecular testing may have variable yield or longer turnaround times.

## Discussion

Our patient was an elderly man with a history of CLL and direct plant and soil exposure who presented with a progressive vesicular lesion and eschar. The diagnosis of invasive cutaneous mucormycosis was achieved by histopathology evaluation. Though antifungal therapy is a key aspect of management, early debridement is essential for source control. Operative findings revealed extensive necrosis of the tendons and muscle, necessitating amputation. He demonstrated clinical improvement thereafter and a cure based on confirmation of clear surgical margins. The source of his infection was hypothesized to be due to direct inoculation from the needle he used to puncture the initial vesicle. An alternate consideration is inadvertent inoculation while gardening, although he had no known trauma to this effect. The favorable factors of this case were the immediate access to the Dermatology and Pathology evaluations for rapid skin biopsy and histopathology results, as well as successful source control.

However, this case posed several challenges, including uncertainty about his cumulative risk for invasive fungal infection (IFI) and the limitations of available diagnostic methods. IFIs with cutaneous manifestations have been well-described in CLL patients on BTK inhibitors, the highest risk class of biologic therapy due to impaired B-cell maturation and function [5-10]. Phosphoinositide 3-kinase (PI3K) inhibitors, such as idelalisib, are moderate to high risk via inhibition of B-cell function and maturation [8]. Anti-CD20 monoclonal antibodies, such as rituximab and obinutuzumab, however, are considered low-moderate risk for IFI despite similar B-cell depleting effects [8]. Impaired humoral immunity and late neutropenia are anticipated effects [8].

To our knowledge, this is the first case report to describe invasive cutaneous mucormycosis in a CLL patient on newer targeted biologic agents, including obinutuzumab, idelalisib, and a BTK degrader trial drug. As the newest anti-CD20 therapy, obinutuzumab has not been associated with fungal infections in large clinical trials, but two cases were later complicated by talaromycosis and *Pneumocystis jirovecii* pneumonia with *Candida krusei* fungemia, respectively [11,12]. In a fatal case of invasive pulmonary *Aspergillus fumigatus* and *Lichtheimia corymbifera* with disseminated disease, the patient was suspected to be at significant cumulative risk due to high-dose corticosteroid use and obinutuzumab-related neutropenia [9]. Several stage 3 clinical trials have identified an increased incidence of *Pneumocystis jirovecii* pneumonia and CMV when idelalisib was added to bendamustine plus anti-CD20 therapy compared to standard of care alone [13-15]. To date, no other published cases have described mucormycosis as a complication of idelalisib. There is insufficient data on the individual risk of IFIs associated with BTK degrader trial drugs. It is possible that these drugs may confer a comparable risk as BTK inhibitors, but more clinical data are needed. Interestingly, our patient was neither neutropenic nor lymphopenic at the time of infection. However, he was known to have low IgA, IgG, and IgM on prior quantitative immunoglobulin panels. This suggests that underlying CLL, associated hypogammaglobulinemia, and the downstream effects of his biologic therapies contributed to his cumulative risk for IFIs [16].

The diagnosis of invasive cutaneous mucormycosis hinged on histopathology, as tissue cultures and fungal NGS were negative, revealing the limitations of current diagnostic methods. Mucorales hyphae can be technically difficult to distinguish from other molds, such as *Aspergillus* spp., partly because the hyphal architecture can be distorted by fixation with paraffin or mishandling of the sample [17]. The degree of inflammatory cells and necrosis can also obscure the detection of fungal elements [18]. Multiple tissue samples may be required to optimize yield. However, intraoperative frozen sections have been shown to be highly sensitive (73-88%) and specific (77-100%) [19,20]. The distinguishing histologic features of *Mucorales* hyphae are broad, ribbon-like, and aseptate branching, staining positive with the addition of Gomori’s methenamine-silver, periodic acid-Schiff, and hematoxylin and eosin (H&E) stains [17]. Conversely, the yield of tissue cultures is low at ~50% due to difficulties with growth based on handling of the sample [3,21]. For similar reasons, the sensitivity of *Mucorales*-specific DNA polymerase chain reaction (PCR) varies based on the target, specimen type, and addition of paraffin fixation [4,17,22]. Despite their reported utility in facilitating early diagnosis, molecular tests may not be readily available depending on the practice setting, have long turnaround times, and have not been externally validated [17].

Clinical cure of mucormycosis is contingent upon early antifungal initiation, surgical source control, and a decrease in immunosuppression. L-AmB at 5-10 mg/kg/day is strongly recommended as the first-line treatment for mucormycosis, while posaconazole (POS) and isavuconazole (ISAV) are moderately recommended [17,23]. Though sometimes used in combination with L-AmB, echinocandins have poor in vitro activity against *Mucorales* spp. as they lack activity against beta-(1,6)-D-glucan synthase, the main component of the fungal cell wall [24]. Our patient was initially treated with a combination of L-AmB and micafungin within two days of admission. However, the efficacy of combination antifungal therapy remains uncertain, given a paucity of randomized controlled studies. The 2014 Infectious Diseases Society of America (IDSA) guidelines on skin and soft tissue infections (SSTIs) outline combination L-AmB or posaconazole (POS) plus an echinocandin as a weak recommendation for *Mucorales* SSTIs [23]. Similarly, the 2019 mucormycosis guidelines by the European Confederation of Medical Mycology (ECMM) marginally support the recommendation for L-AmB with POS, L-AmB with caspofungin, and L-AmB with micafungin or anidulafungin [17]. In cases of disseminated or refractory disease or clinical deterioration, POS or ISAV may be added to L-AmB [17].

A more granular appraisal of the data is necessary to better assess the efficacy of combination antifungal therapy: (a) drug choice (L-AmB with echinocandin or L-AmB with POS or ISAV); (b) timing (immediately after diagnosis versus salvage); (c) site of disease (pulmonary, cutaneous, rhino-orbital-cerebral, or disseminated). Improvement in clinical outcomes after combination L-AmB and caspofungin has been demonstrated in murine models, but not in most retrospective clinical studies [24-26]. In a retrospective study by Reed et al., improved survival was seen with L-AmB and caspofungin compared with monotherapy in a sample of 41 diabetic patients with rhino-orbital-cerebral disease (OR: 10.9, 95% CI: 1.3-$\infty$, p = 0.02) [25]. However, larger retrospective studies of patients with hematologic malignancies did not demonstrate a significant difference in survival [27,28]. This was demonstrated in a propensity score adjustment by Kyvernitakis et al., which included a majority of sinopulmonary cases [28].

Favorable results can also be seen in murine models of L-AmB and POS or L-AmB and ISAV, but not statistically significant in clinical studies [28-31]. A neutropenic mouse model of L-AmB and ISAV suggested drug synergy with a significant reduction in tissue fungal burden at day four (2.0-3.5 log reduction vs. 1.0 reduction for either drug) [30]. Survival was significantly improved to 80% with this combination compared with 50% for either drug, but the follow-up interval was brief at 21 days [30]. To date, there is scant clinical data on L-AmB with ISAV, but a recent study, which included three cases, did not demonstrate a significant improvement in survival [29]. Retrospective studies of L-AmB with POS have also been concordant with this finding [27-29].

Regarding the timing of combination therapy, murine and pre-clinical studies have mostly studied the outcomes of this approach early in diagnosis. There is only one study to date that sought to differentiate outcomes of early versus salvage combination therapy, but did not support a difference in six-week mortality (OR: 1.44, 95% CI: 0.29-7.23, p = 0.66 compared with OR: 2.15, 95% CI: 0.61-7.66, p = 0.24, respectively) [29]. Since 88% of early combination therapy occurred in cases with disseminated disease, it is possible that the severity of disease was a confounder [29]. Disseminated or sinopulmonary disease has been identified as a risk factor for increased mortality [28,31]. Therefore, the site of disease should also be considered in future clinical studies on combination therapy.

## Conclusions

This is the first known case to describe invasive cutaneous mucormycosis in a patient with CLL and newer targeted biologic agents (BTK degrader, obinutuzumab, and idelalisib). This case highlights lesser-known, potential contributions of newer targeted biologic agents to the net state of immunosuppression, warranting further study. To facilitate timely diagnosis and limb-sparing outcome, a low index of suspicion for invasive mucormycosis and a multimodal diagnostic approach with histopathology, culture, and/or molecular testing are essential. While available for other molecular methods, more data are warranted to evaluate the diagnostic yield of metagenomic next-generation sequencing in invasive mucormycosis based on specimen type and host factors. This is essential as tissue biopsy or culture may be precluded by patient comorbidities. Nevertheless, early diagnosis was possible for our patient with histopathology. Though combination antifungal therapy was used upfront in our case, the utility of this approach is less supported by available clinical data. Ultimately, clinical cure was achieved with prompt initiation of antifungal therapy and timely surgical amputation.
